# Association of Liver Biomarkers With Jaundice in Patients With Gastrointestinal Complications: An Observational Retrospective Cohort Study

**DOI:** 10.7759/cureus.75583

**Published:** 2024-12-12

**Authors:** Muhammad Hassan Raza, Awais Mustafa, Muhammad Amir, Muhammad Khurram Saleem, Hifza Ishtiaq, Amna Akbar, Mohammad Saleem Khan

**Affiliations:** 1 Gastroenterology, Russells Hall Hospital, Dudley, GBR; 2 Gastroenterology, Primary and Secondary Healthcare Department, Faisalabad, PAK; 3 Medicine, Midland Doctors Medical Institute, Muzaffarabad, PAK; 4 General Internal Medicine, University Hospitals Bristol and Weston NHS Foundation Trust, Bristol, GBR; 5 Medicine, Abbas Institute of Medical Sciences, Muzaffarabad, PAK; 6 Emergency Medicine, Alexandra Hospital, Redditch, GBR; 7 Medicine, District Headquarter (DHQ) Teaching Hospital, Kotli, PAK

**Keywords:** biomarker, enzyme, gastroenteritis, jaundice, lft, liver

## Abstract

Introduction: Jaundice is a state of disease in liver function often along with gastrointestinal (GI) complications primarily characterized by hyperbilirubinemia. Other tests regarding liver function are often used besides bilirubin as during any liver complications biomarkers such as alanine aminotransferase (ALT), serum glutamic pyruvic transaminase (SGPT), aspartate aminotransferase (AST), serum glutamic-oxaloacetic transaminase (SGOT), alkaline phosphatase (ALP), and many other biomarkers even total protein metabolism might get disrupted and get released in the blood. However, no comparison or association among those biomarkers was determined in a cohort.

Method: In this observational retrospective cohort study, we investigated the levels of different liver biomarkers and tried to compare their differences and correlations in jaundice patients with GI complications. We examined nine different biomarkers where, primarily, the jaundice was confirmed through the total bilirubin test and further categorized and analyzed them.

Result: Among the 91 patients of the study cohort after bilirubin (n=91) and conjugated bilirubin (n=88) levels, we found that enhanced globulin (n=79) levels were the most dominant among the cohort followed by unconjugated bilirubin (n=76), albumin (n=72), and total protein (n=71). On the other hand, a high ALP level was determined in the lowest number of the population (n=22). Regarding the category of men and women, similar findings were observed with a slight variation as the number of included female patients was approximately six times higher than that of male patients. In the case of age groups, similar observations were determined with some slight variation, as most of the patients were found to be over 40 years of age.

Conclusion: Besides bilirubin, globulin can be a promising biomarker to diagnose jaundice, especially in patients with GI complications. It may also help to indicate the presence of undiagnosed or unsuspected jaundice in patients with gastrointestinal problems.

## Introduction

Jaundice is a clinical condition that is usually associated with complications related to the liver, sometimes bile duct, or other pathophysiological factors where serum bilirubin levels are elevated and create a condition of hyperbilirubinemia causing yellow discoloration of the mucus membrane, eyes, and skin. Serum bilirubin is produced from the heme-ring breakdown of the red blood cells metabolized by the liver. The liver usually governs the metabolism of bilirubin through conjugation or excretion. This metabolism process is hampered in the case of jaundice which leads the patients toward hyperbilirubinemia [1,2]. Acute jaundice frequently indicates a significant underlying health issue and arises from both intrahepatic and extrahepatic sources. Research has revealed that intrahepatic disorders account for the majority of acute jaundice cases in adults, with viral hepatitis, alcoholic liver disease, and drug-induced liver injury being the primary culprits. The remaining cases of acute jaundice are attributed to extrahepatic causes, including gallstone disease, hemolysis, and malignancy [3-5].

Gastrointestinal (GI) complications frequently occur in patients with jaundice. Satiety, abdominal pain, diarrhea, vomiting, and nausea are some common symptoms related to GI problems in jaundice. However, GI damage and bleeding can sometimes occur in severe cases along with edema, anemia, and even neurological disorders such as kernicterus, which may ultimately lead patients to coma or death in jaundice [2,6-8].

Since jaundice may not be readily apparent through physical examination, investigation of the serum bilirubin and liver biomarkers is required to confirm the diagnosis. Besides, serum bilirubin, alanine aminotransferase (ALT) or serum glutamic pyruvic transaminase (SGPT), aspartate aminotransferase (AST) or serum glutamic-oxaloacetic transaminase (SGOT), alkaline phosphatase (ALP) are measured [9-11]. However, no study assessed the correlation and differences among the liver biomarkers in jaundice patients with hyperbilirubinemia. Therefore, in this study, we investigated the liver biomarkers to determine the levels and compare their correlation in jaundice patients with GI complications.

The primary objective of this study was to determine the presence of elevated levels of nine different biomarkers in jaundice patients with GI complications where jaundice was initially confirmed by measuring the elevated total bilirubin (TB) level. The secondary objective was to determine the levels of biomarkers in different categories (i.e. gender and age) of patients. 

## Materials and methods

Study population

The study was conducted at the Abbas Institute of Medical Sciences, Muzaffarabad, AJK, Pakistan. The ethical approval number was AIMS/8816/2024. Data for this retrospective study was collected from the hospital register from April 2024 to September 2024. A total of 558 participants’ data were primarily checked from the hospital register in this cohort study.

Inclusion and exclusion criteria

Inclusion and exclusion criteria were maintained before the data collection for this study. According to the inclusion criteria, jaundice patients who were confirmed through physiological examination by the physicians and hyperbilirubinemia by testing TB and had non-severe GI complications such as satiety, abdominal pain, diarrhea, vomiting, and nausea and also did certain liver function tests including conjugated bilirubin (CB), unconjugated bilirubin (UCB), ALT, or SGPT, AST, SGOT, ALP, Gamma-glutamyl transferase (GGT), total protein (TP), albumin and globulins were included in the study. Both male and female patients were considered eligible for this study. No age restriction was maintained. As per the exclusion criteria, patients with other metabolic or chronic diseases such as diabetes, cancer, cardiovascular diseases, kidney or renal disease, or other hospital patients who did not meet the inclusion criteria accurately or healthy individuals were excluded from the study. 

Data collection

Data was collected regarding the levels of biomarkers from the hospital registers. We primarily collected the patient's ID, age, and gender followed by the data of TB, CB, UCB, ALT (SGPT), AST (SGOT), ALP, GGT, TP, albumin, and globulin levels respectively along with the specific unit in which the result was reported and the reference value. The biomarkers were tested using serum samples of the patients. Primarily through venipuncture blood samples were collected from patients. Then through the centrifugation method serum was separated from the hematocrit and the biomarkers were tested and stored if they remained at -20°C. Automatic biochemistry analyzer Dimension® EXL™ 200 (Siemens Healthineers-Laboratory Diagnostics, Germany) was used. 

Statistical analyses

Data collection, graphical representations, and statistical analyses were done using Microsoft Excel (Windows 10; Microsoft Corporation, Redmond, US) and IBM SPSS Statistics for Windows, Version 16 (Released 2007; IBM Corp., Armonk, New York, United States). The mean, standard deviation (SD), data frequency, and percentages were determined. The frequencies and the ratios of the patients with elevated biomarker levels were presented in the bar chart. 

## Results

Patient demographics

We included 91 confirmed jaundice patients who had GI complications as well through physical examination and TB test where all the patients were high in TB (>reference value, 1.0 mg/dL). Among them, male patients were less in number (n=12, 13.19%) as compared to female patients (n=79, 86.81%). The mean age was 57.62 ± 20.88 (Table 1).

**Table 1:** Patient demographics. SD: standard deviation, CVD: cardiovascular disease

Variables	Frequency, n	Percentage, %
Total population	91	100
Male	12	13.19
Female	79	86.81
Age (mean ± SD)	57.62 ± 20.88	-
Body mass index (BMI) (mean)	22.89	-
Blood pressure (BP) (mean)	123-84 (mm Hg)	-
Diabetes	No	-
HbA1c (mean ± SD)	5.18 ± 0.87	-
Cancer (current/previous)	No	-
CVD (current/previous)	No	-
Kidney/renal disease (current/previous)	No	-
Ethnicity	Asian (Pakistani)	-
Total bilirubin (>Ref range, 1.0mg/dL)	91	100%

Comparison of patient frequencies with biomarkers

**Figure 1:**
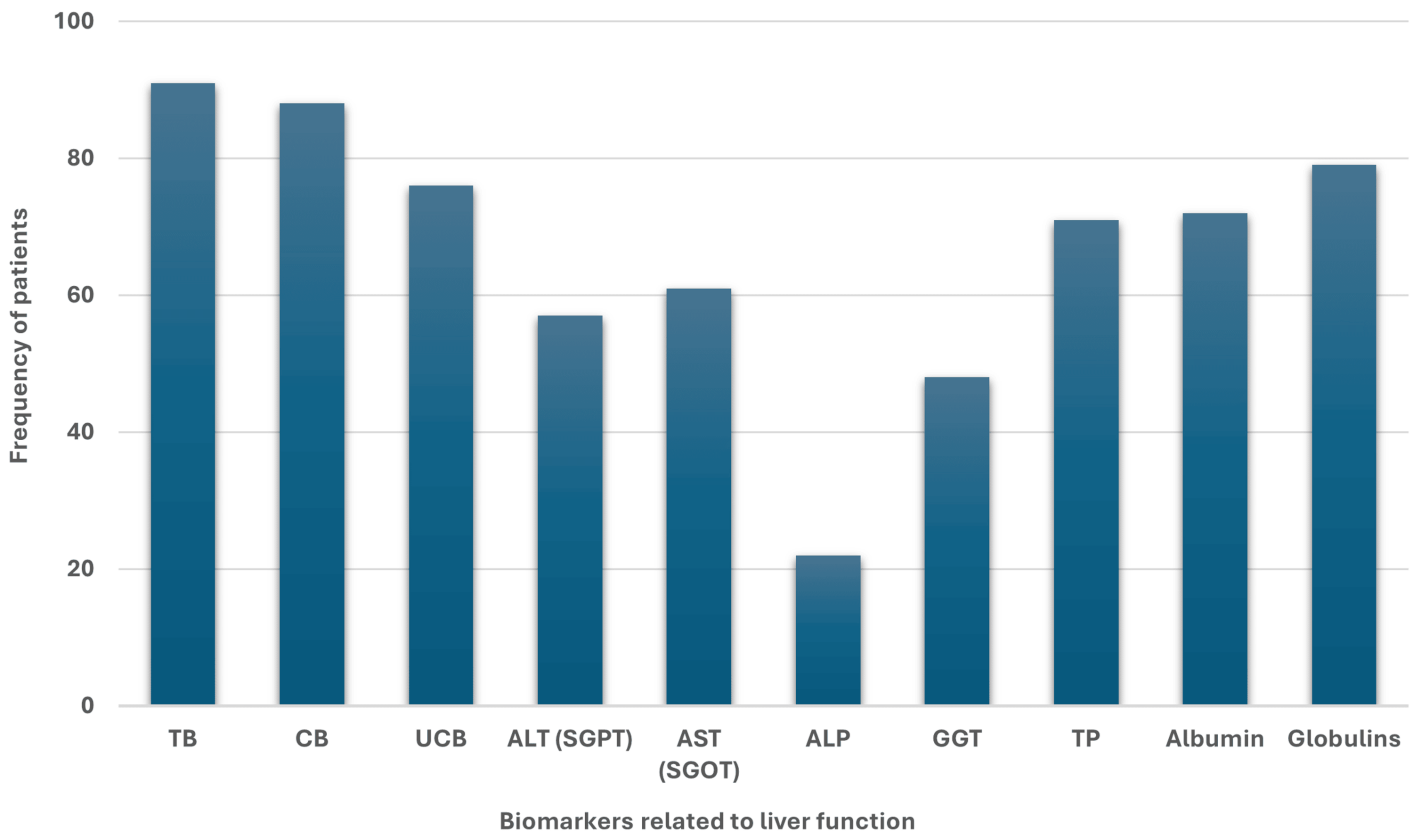
Frequency of jaundice patients with higher (>Ref value) levels of liver biomarkers related to liver function. TB: total bilirubin, CB: conjugated bilirubin, UCB: unconjugated bilirubin, ALT: alanine aminotransferase, SGPT: serum glutamic pyruvic transaminase, AST: aspartate aminotransferase, SGOT: serum glutamic-oxaloacetic transaminase, ALP: alkaline phosphatase, GGT: gamma-glutamyl transferase, TP: total protein

Comparing the data of overall patients, we found that the frequency of patients was the highest (n=79, 86.81%) with high (>Ref value, 3.5 g/dL) globulin levels after TB (n=91, 100%) and CB (n=88, 96.70%). Even the frequency of the UCB (n=76, 83.51%) was lower among patients as compared to the globulins followed by albumin (n=72, 79.12%) and TP (n=71, 78.02%). Again, the frequency of the ALP was the lowest (n=22, 24.17%), followed by the AST (n=61, 67.03%), ALT (n=57, 62.63%), and GGT (n=68, 52.74%) (Figure 1).

**Figure 2:**
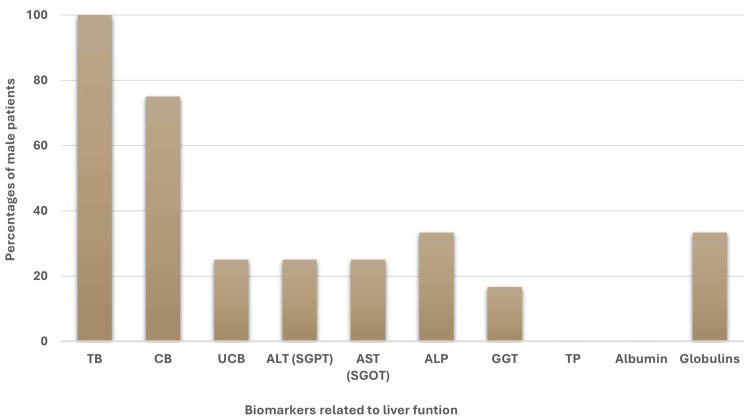
Percentage of male jaundice patients with higher (>Ref value) levels of liver biomarkers related to liver function. TB: total bilirubin, CB: conjugated bilirubin, UCB: unconjugated bilirubin, ALT: alanine aminotransferase, SGPT: serum glutamic pyruvic transaminase, AST: aspartate aminotransferase, SGOT: serum glutamic-oxaloacetic transaminase, ALP: alkaline phosphatase, GGT: gamma-glutamyl transferase, TP: total protein

Interestingly, in the case of male patients, after TB (n=12, 100%) and CB (n=9, 75%) globulins and ALP were found as the highest and same frequencies (n=4, 33.33%) and the frequency of GGT (n=2, 16.66%) was the lowest, whereas higher levels of TP and albumin were absent among male patients (Figure 2).

**Figure 3:**
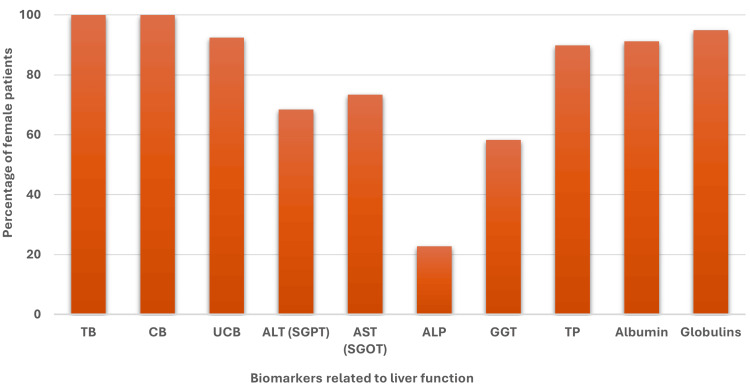
Percentage of female jaundice patients with higher (>Ref value) levels of liver biomarkers related to liver function. TB: total bilirubin, CB: conjugated bilirubin, UCB: unconjugated bilirubin, ALT: alanine aminotransferase, SGPT: serum glutamic pyruvic transaminase, AST: aspartate aminotransferase, SGOT: serum glutamic-oxaloacetic transaminase, ALP: alkaline phosphatase, GGT: gamma-glutamyl transferase, TP: total protein

 However, in the case of female patients, the same observation was determined as the overall cases (Figure 3).

**Figure 4:**
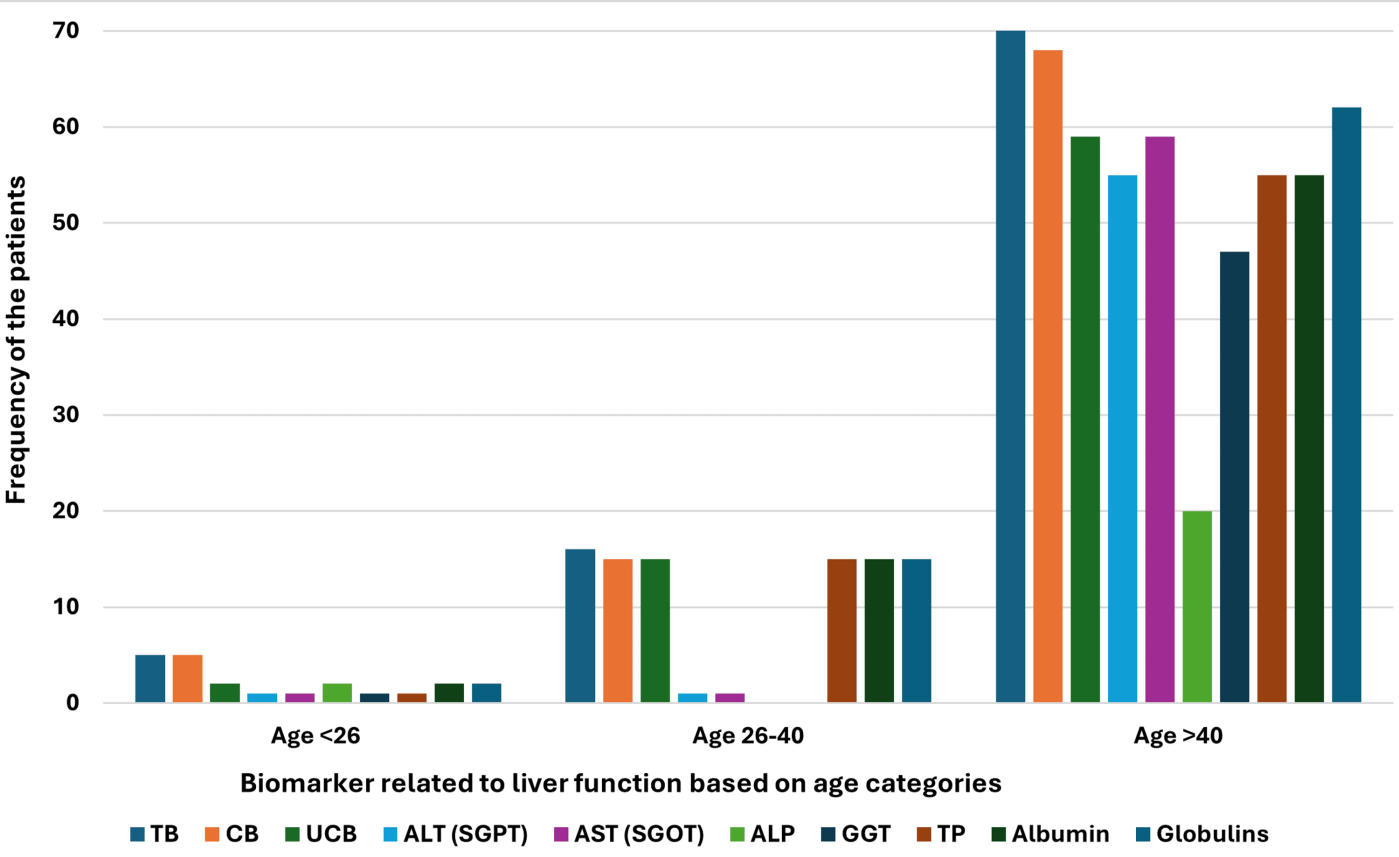
Frequency of the jaundice patients with higher (>Ref value) levels of liver biomarkers related to liver function categorized by the age group. Here are three major age groups: I) Age <26 years, II) Age 26-40 years and III) Age >40 years. TB: total bilirubin, CB: conjugated bilirubin, UCB: unconjugated bilirubin, ALT: alanine aminotransferase, SGPT: serum glutamic pyruvic transaminase, AST: aspartate aminotransferase, SGOT: serum glutamic-oxaloacetic transaminase, ALP: alkaline phosphatase, GGT: gamma-glutamyl transferase, TP: total protein

In age-based categories, we observed that age group III (age>40) was the highest in number (n=70) in our cohort and thus showed a similar manner in the frequencies of the patients with higher levels of biomarkers with slight changes. Interestingly, in age group III, AST was higher (n=59, 84.28%) in patient numbers as compared to albumin (n=55, 78.57%) and TP (n=55, 78.57%) where the number matched with ALT (n=55, 78.57%). However, the globulin (n=62, 88.57%) was the highest in age group III after TB (n=70, 100%) and CB (n=68, 97.14%). Age group II (age 26-40) was lower in number (n=16) and showed slight dissimilarities where the frequencies of patients with higher levels of CB, UCB, TP, albumin, and globulins were the same (n=15, 93.75%). However, no patients with higher ALP and GGT were detected. In age group I (<26) there were only five patients; however, the frequencies of TB (n=5, 100%), and CB (n=5, 100%) were the highest, and the UCB, ALP, albumin, and globulin were present in higher levels in the same number of patients (n=2, 40%) (Figure 4).

Comparison of the biomarker levels

While comparing the biomarker levels in different groups (i.e. male, female, and age) we observed a pattern that in TB, CB, UCB, TP, albumin, and globulin the levels were higher in female patients and age group III as compared to the levels of male, Age group I and II. On the other hand, ALT levels were higher in lower in female patients and age group III as compared to the levels of male patients and age groups I and II. AST, ALP, and GGT were higher in male patients and age group I than in female patients and age group III. However, in age group II no ALP and GGT were determined, and AST was the lowest in level (Table 2) 

**Table 2:** The mean levels of liver biomarkers in jaundice patients with higher (>Ref value) biomarker levels categorized by the age groups LB: liver biomarkers, TB: total bilirubin, CB: conjugated bilirubin, UCB: unconjugated bilirubin, ALT: alanine aminotransferase, SGPT: serum glutamic pyruvic transaminase, AST: aspartate aminotransferase, SGOT: serum glutamic-oxaloacetic transaminase, ALP: alkaline phosphatase, GGT: gamma-glutamyl transferase, TP: total protein, NA: not applicable, as no patients were found in that category.  The values are expressed as the mean value. The reference values for total bilirubin, conjugated bilirubin, unconjugated bilirubin, ALT, AST, ALP, GGT, total protein, albumin, and Globulins were up to 1.0 mg/dL, 0.30 mg/dL, 1.0 mg/dL, 45 U/L, 35 U/L, 116 U/L, 55 U/L, 8.50 g/dL, 5.20 g/dL, and 3.50 g/dL, respectively.

Liver biomarkers	Overall LB levels	LB levels (Male)	LB levels (Female)	LB levels (Age group I, <26 years)	LB level (Age group II, 26-40 years)	LB Level (Age group III, >40 years)
TB (mg/dL)	29.43	1.48	33.68	1.42	9.63	35.96
CB (mg/dL)	30.19	1.16	33.5	0.94	10.1	36.78
UCB (mg/dL)	34.76	1.56	36.13	1.6	10.1	42.16
ALT (SGPT) (U/L)	72.01	85	71.29	111	88	71.01
AST (SGOT) (U/L)	66.08	84.66	65.12	134	55	65.11
ALP (U/L)	231.5	329.5	209.72	516.5	NA	203
GGT (U/L)	116.18	243.5	110.65	368	NA	110.82
TP (g/dL)	43.9	NA	43.9	8.9	16.9	51.9
Albumin (g/dL)	40.9	NA	40.9	5.9	14.4	49.4
Globulins (g/dL)	35.93	4.05	37.64	4.3	12.5	42.63

## Discussion

Jaundice is widely classified into three categories i.e. pre-hepatic, hepatic, and post-hepatic or cholestatic. Pre-hepatic jaundice develops when the liver cannot conjugate bilirubin as quickly as it is produced; typically, the liver can process up to six times the normal daily amount before bilirubin levels in the plasma increase. In this condition, serum transaminases such as AST, ALT, and ALP remain within normal ranges [12-14]. Several factors can lead to this, including ineffective erythropoiesis, reabsorption of hematomas, extensive blood transfusions due to the reduced lifespan of transfused red blood cells, homozygous sickle cell disease, and thalassemia major [13,15]. Unconjugated hyperbilirubinemia is also observed in conditions where bilirubin conjugation is impaired, such as Gilbert's syndrome and Crigler-Najjar syndrome. Again, hepatic jaundice occurs when hepatocytes fail to effectively secrete conjugated bilirubin into the bile canaliculi. In this type, serum transaminases are often elevated due to the underlying liver condition [12,16,17]. Common hepatic causes include viral hepatitis, alcohol-related liver disease, primary biliary cirrhosis, drug-induced jaundice, and alcoholic hepatitis. Post-hepatic or cholestatic jaundice is frequently caused by biliary obstruction, where insufficient bile reaches the duodenum. The most frequent obstruction in the biliary system is due to gallstones or choledocholithiasis. Other causes of post-hepatic jaundice include biliary strictures, trauma, inflammatory processes, malignancies, and external compression, such as pancreatic cancer [12,18,19]. From the pathophysiology, we get an indication that transaminase biomarkers such as AST, ALT, and ALP are not increased during jaundice simultaneously rather they require specific conditions to elevate. This study also found similar observations where a large number of jaundice patients had normal ranges of AST, ALT, and ALP.

UCB was reported to be prevalent mostly in cases of increased production, reduced hepatic uptake, and diminished conjugation of bilirubin or in neonatal jaundice. Additionally, several genetic conditions can cause unconjugated hyperbilirubinemia, including Gilbert syndrome, Crigler-Najjar syndrome types I and II, and inherited disorders that result in hemolytic anemia [16,17,20]. Therefore, in some other causes of jaundice, it may produce slightly less as compared to CB. We observed that in all the categories, the frequency of raised CB levels in jaundice patients was higher than raised UCB levels, which can be understood by this phenomenon. Besides, albumin is usually bound to UCB [20]. Therefore, the elevation of UCB also prompts the level of albumin in the serum. Therefore, we also detected the frequency of elevated UCB and albumin in jaundice patients to be almost similar. 

Nevertheless, the frequency of elevated levels of globulins was found higher in almost every category of jaundice patients after TB. Previously, transferrin (TRF) which is a β-globulin was found to be a significant marker for the diagnosis of pathological form of jaundice [21]. In this study, we observed that elevated levels of globulins were the most prevalent among the jaundice patients just after TB and CB as compared to all other biomarkers. Another study also confirmed that serum globulin is a diagnostic marker providing important information regarding hepatic function [22]. 

We observed that in our study cohort, most of the patients were female and aged persons. Women were previously found prevalent in multiple studies in the case of obstructive jaundice. Among them, the patients were older as well which supports our findings [23,24].

Limitations of the study

As this was a retrospective cohort and single-centered study, we only analyzed and compared the biomarker levels in patients but could not enhance the sample size and population variation maintaining our inclusion and exclusion criteria. Besides, we could not do multivariate analyses and check the disease effect on biomarkers separately based on age and sex maintaining an equal sample size. We also could not rigorously determine the molecular mechanisms of globulins or select any specific globulin that can be a particular biomarker for jaundice, rather than observe a frequency-based association between jaundice patients and globulins level. Therefore, we cannot claim that globulin can replace TB or CB yet.

## Conclusions

We investigated that globulins are significantly prevalent in jaundice patients and most of the patients were found to be female and older. These indicate that globulins may be a remarkable biomarker for diagnosing and prognosis of jaundice. Besides, women and older individuals should be careful regarding their lifestyle and liver health to avoid jaundice and other liver-related complications. Although TB and CB were found to be the “gold standard” for the diagnosis of jaundice, unsuspected or asymptomatic jaundice, especially in patients with GI complications, globulin detection may be a remarkable indicator. However, more rigorous research is required to find the mechanism and association of globulins especially any specific globulin with GI complication-associated jaundice.
